# Progress in the implementation of massively parallel sequencing for forensic genetics: results of a European-wide survey among professional users

**DOI:** 10.1007/s00414-021-02569-0

**Published:** 2021-04-13

**Authors:** Theresa E. Gross, Jan Fleckhaus, Peter M. Schneider

**Affiliations:** 1 Institute of Legal Medicine, Faculty of Medicine and University Clinic, University of Cologne, Melatengürtel 60/62, 50823 Cologne, Germany; 2 Present Address: Hessisches Landeskriminalamt, Wiesbaden, Germany

**Keywords:** Next-generation sequencing, DNA phenotyping, Forensic DNA casework, Online survey

## Abstract

A European-wide online survey was conducted to generate an overview on the state-of-the-art using massively parallel sequencing (MPS) platforms for forensic DNA analysis and DNA phenotyping among forensic practitioners in Europe. The survey was part of the dissemination activities of the “VISible Attributes through GEnomics – VISAGE” Horizon 2020 funded European research project [30], in preparation of a series of educational training activities. A total of 105 replies from 32 European countries representing participants from police, governmental, academic, and private laboratories providing professional services in the field of forensic genetics were included in the final analysis. Of these, 73% already own an MPS platform or are planning to acquire one within the next 1–2 years. One-third of the participants have already carried out MPS-based STR sequencing, identity, or ancestry SNP typing. A total of 23–40% of participants are planning to explore all FDP applications showing the overall very high interest in using MPS for the whole range of forensic MPS markers and applications. About 50% of the participants have previously gathered experience using forensic DNA phenotyping (FDP) markers based on conventional (i.e., not MPS-based) DNA typing methods. A total of 55% of the participants have attended training on the general use of MPS technology, but 36% have received no training whatsoever. Accordingly, 90% have expressed high or medium interest to attend training on the analysis and interpretation of DNA phenotyping data for predicting appearance, ancestry, and age. The results of our survey will provide valuable information for organizing relevant training workshops on all aspects of MPS-based DNA phenotyping for the forensic genetics scientific community.

**Supplementary Information:**

The online version contains supplementary material available at 10.1007/s00414-021-02569-0.

## Introduction

The technology used for short tandem repeat (STR) marker typing based on multiplex PCR followed by capillary electrophoresis is well established in all forensic DNA laboratories. In contrast, platforms for massively parallel sequencing (MPS) require a different analytical workflow, as well as novel and currently not widely available forensic bioinformatic resources due to the massive amount of data generated; i.e., millions of reads per sample can be obtained from a single sequencing experiment [1–3]. In addition to STRs, different types of forensic markers can be analyzed with MPS technology such as single-nucleotide polymorphisms (SNPs), mitochondrial DNA, DNA methylation patterns, and mRNA gene expression for body fluid identification. These markers are particularly useful for forensic DNA phenotyping (FDP)—the DNA-based prediction of appearance (eye, hair, and skin color), biogeographic ancestry, and age [4, 5]. The “VISible Attributes through GEnomics – VISAGE” Consortium is developing MPS tools for analysis of FDP markers to allow for prediction of appearance, ancestry, and age of unidentified contributors from biological stains. These tools are designed to enable forensic casework laboratories to implement these innovative approaches into their repertoire of routine applications for investigating casework samples [6–8].

The primary results of MPS analysis cannot be readily understood and need several layers of software tools and user interfaces for interpretation. Furthermore, the probabilities leading to the construction of composite sketches require a good understanding of the structure and type of data as well as underlying genetic models and the framework defining the range of predicted features regarding appearance, age, and ancestry. In the wake of a survey carried out in 2017 on the availability of MPS technology and applications among 33 forensic DNA labs from 25 countries [9], the VISAGE Consortium needed to compile more extensive data to prepare for the dissemination of the tools developed within this project. Therefore, we have carried out a detailed European-wide inquiry during the year 2019 to collect up-to-date information about the technical standards of MPS and the availability of laboratory and bioinformatic resources in the end user facilities, as well as about the knowledge and understanding of forensic DNA phenotyping approaches and the corresponding interpretation framework.

This report provides all details about the design and format of this survey and the selection of participants. The main part of the report presents the MPS technology-related information collected from participants, followed by conclusions for subsequent activities, i.e. the development of curricula for user training and educational workshops to provide detailed guidance for the practical implementation of the tools developed by the VISAGE Consortium for MPS-based DNA phenotyping.

## Methods

The purpose of this survey among forensic practitioners was (i) to assess the level of implementation and use of MPS platforms including their various forensic applications, and to identify (ii) the level of individual knowledge and previous practical experience, as well as (iii) training and education needs in regard to forensic DNA phenotyping—the DNA-based prediction of appearance (eye, hair, and skin color), biogeographic ancestry, and age.

We chose the format of an interactive online survey as this enabled us to reach out to all potential participants via email. We have used the online platform UmfrageOnline, a Swiss website offering an easy-to-use and interactive design for online surveys [10]. The questionnaire was divided into the following five different sections: (A) background information, (B) equipment and personnel, (C) forensic DNA phenotyping applications, (D) education and training, and (E) final comments, comprising a total of 19 questions. A visual representation of the online survey is available as electronic supplement 1.

Approximately 400 invitations were sent out to members of the European Network of Forensic Science Institutes (ENFSI) DNA Working Group [11], participants of the annual German DNA Profiling (GEDNAP) proficiency test [12], and the members of the European DNA Profiling (EDNAP) Group, a Working Group of the International Society for Forensic Genetics (ISFG) [13]. We did not have direct access to the mailing list of the GEDNAP laboratories due to the data protection policy of the organizing institute of the GEDNAP proficiency test; thus, recipients were not disclosed individually. Accordingly, some recipients may have been included in several of these groups and we have estimated the rate of duplicate invitations to be about 20–30% of the approx. 400 total invitations. Furthermore, about 20% of non-European recipients were included in the initial email dispatch. These were identified based on the submitted answers regarding the country of residence and removed from the data set for this report.

## Results and discussion

### Final dataset

We received replies from a total of 141 laboratories of which 128 labs completed the survey to full extent. We have cleaned our dataset to reach a total of 105 replies by filtering out the answers of several participants from outside of Europe, multiple participations from the same individual, and multiple participations from the same institution by different employees. Given the fact that approximately 300–350 potential participants were addressed (the exact number remains unknown because of the non-disclosure of the private mailing list from the GEDNAP proficiency test), the overall response rate was 25–30%.

#### Part A: Background information

First, we asked participants for some background information on their laboratory and themselves including their country of residence, which organization their lab is affiliated with and if it is a member of the ENFSI DNA Working Group, their role within the lab, and their educational background. Furthermore, laboratories were asked to estimate the amount of criminal casework in regard to the total amount of routine casework, if applicable.

##### Country of residence and organization

The participants came from institutions residing in 32 different European countries. Almost one-third (28.6%) were from Germany (see Table 1 and S1). This is mostly due to the fact that forensic DNA typing in Germany is carried out by a variety of police, academic, and private laboratories and that the dissemination of the survey was also done via the mailing list of the GEDNAP proficiency test, which traditionally has a large number of German participants. Other populous European countries such as Italy, Spain, Poland, and France were represented by 4–9 different participants. It might thus be argued that the survey results could be biased with an overrepresentation of German labs. However, due to the federal structure of Germany consisting of 16 states that are independent regarding police affairs, the participating labs have a heterogeneous background, coming from governmental, academic, and commercial entities exhibiting a diversity of organizational structures.

**Table 1:** European survey participants by numbers and percentages

Country	Sample	Percent
Albania	1	1.0%
Austria	2	1.9%
Belgium	4	3.8%
Bosnia and Herzegovina	1	1.0%
Croatia	2	1.9%
Czech Republic	3	2.9%
Denmark	1	1.0%
Estonia	1	1.0%
Finland	1	1.0%
France	4	3.8%
Germany	30	28.6%
Greece	4	3.8%
Hungary	1	1.0%
Ireland	1	1.0%
Italy	9	8.6%
Latvia	2	1.9%
Lithuania	1	1.0%
Luxembourg	1	1.0%
Macedonia	2	1.9%
Montenegro	1	1.0%
Netherlands	4	3.8%
Norway	2	1.9%
Poland	4	3.8%
Portugal	2	1.9%
Romania	3	2.9%
Serbia	1	1.0%
Slovakia	1	1.0%
Slovenia	1	1.0%
Spain	7	6.7%
Sweden	1	1.0%
Switzerland	4	3.8%
UK	3	2.9%

The majority of survey participants were either from police/governmental or academic laboratories (about 40% each) while about a sixth of replies resulted from private laboratories (S2). Slightly more than half of all responses from European laboratories were obtained from non-ENFSI DNA Working Group members (S3).

##### Position and education of participants

In addition to general information about their laboratory, participants were also asked to provide details about their own role within the lab and their academic or educational background (S4). More than 90% of participants were holding at least a master’s degree or higher and were either head of the corresponding laboratory or employed as a scientist or reporting officer. This result is a logical consequence of our survey dissemination strategy as usually the lab director’s name is given in the ENFSI, EDNAP, or GEDNAP mailing lists. As our survey was aiming to reach the scientists in charge of research and the decision-making process, these results confirm that we had reached our target group.

##### Contribution to criminal casework

At the end of this section, participants were asked about the involvement of their lab in criminal casework, and if so, to give an estimate of the amount of crime cases in regard to the overall casework in the respective laboratory. A clear majority of contributing laboratories (76.2%) are spending between 50 and 100% of their time for performing and reporting casework in the context of criminal investigations (S5). One-tenth of laboratories—only private or academic laboratories within this survey—are not involved in criminal casework while all contributing police or governmental laboratories (43 in total) are obtaining at least half of their routine casework from criminal investigations. Not surprisingly, the majority of laboratories exclusively performing criminal casework are police or governmental laboratories (30/37, about 81%), whereas most academic laboratories usually spend about 50% of their time on casework concerning criminal investigations. This outcome confirms once again that the survey was distributed within the correct target group.

#### Part B: Equipment and personnel

The second part of the VISAGE survey interrogated the participants about the availability and types of MPS platforms, additional equipment, analysis software for MPS data, and dedicated personnel for operation of MPS platforms, data analysis, and interpretation in their own laboratory.

##### Ownership of massively parallel sequencing equipment

We were surprised to learn that already 46% of participants own a platform for massively parallel sequencing, and another 26.7% have either ordered a platform or are planning to do so within the next 24 months (Table 2, S6). Hence, a clear majority of participants has made a decision to adopt this new technology into their methodological portfolio.

**Table 2:** Composition of survey participants and previous experience with MPS platforms

	ENFSI member		MPS ownership		Planned purchase within		No plans	Type of instrument		
Type of laboratory	Yes	No	> 2 years	< 2 years	12 months	1–2 years		MiSeq	Ion PGM	Other
								MiSeqFGx	Ion S5	
Police/governmental	37	6	14	5	2	12	10	12	10	
Academic	9	32	17	6	2	6	10	13	18	3
Private	1	17	3	1	1	5	8	3	2	
Other	1	2		2			1	2		1
Total	48	57	34	14	5	23	29	30	30	4

The type and manufacturer of the instruments available to the 48 laboratories who already own a platform are shown in S7. Overall, 64 platforms are available; i.e., about one-third (16 laboratories) already own or have access to more than one instrument. The vast majority of instruments are either Verogen’s MiSeq FGx or Thermo Fisher’s Ion S5 platforms. Other MPS platforms available are Illumina’s HiSeq or NextSeq, and Thermo Fisher’s Ion Proton instruments. Availability of accessory equipment, such as pipetting robots, robots for library preparation, and chip loading, as well as data storage systems is shown in S8: 33 labs have access to robotic platforms for sample and library preparation and 10 labs have both a robot and a network data storage system.

##### Analysis software for massively parallel sequencing

The most crucial element for MPS-based DNA typing in addition to the technical competence in sample and library preparation is the availability of suitable software for secondary and tertiary analysis of sequencing data and their respective interpretation.S9 provides a summary of the types of software available to the labs already owning an MPS platform (*n* = 48). More than half of those labs gave more than one answer to this question indicating that several different software tools are in use, whereas 23 labs use only one type of software, which is mostly the one provided by the platform manufacturer (18/23). A clear majority of labs using more than one software tool are relying on the manufacturer’s software plus online resources, while three or more tools including the latter two are applied in 12 labs with either an in-house bioinformatic pipeline or an open source software tool.

Other software tools used by participants including open source and online resources are IGV browser, STRait Razor, FDS Tools, toaSTR, Yleaf, BWA, SamTools, GATK, Genogeographer, FROG-kb, and Ingenuity® Variant Analysis™ [14–20].

##### Dedicated personnel for massively parallel sequencing

Aside from information about the technical circumstances of introducing MPS in each lab, we received answers from 93 participants (12 did not provide an answer) regarding the availability or plans to have dedicated personnel for operating the MPS platform. S10 displays the current or planned use for three different types of personnel and the number of tasks they have been (or will be) assigned to. Here, 39% and 48% of participants already have either a dedicated technical assistant or a scientist, respectively, to handle this technology. On the other hand, specific programming competence with the presence of a bioinformatics expert is available only to 19% of the labs.

The breakdown of tasks is shown in S11. Here, the three different types of personnel (scientist, bioinformatics expert, technical assistant) are shown separately with their types and numbers of tasks assigned. While technical assistants and bioinformatics experts have only one task in the majority of labs, i.e. library preparation and data analysis, respectively, almost 40% of the scientists have to handle two or three different tasks, in particular including phenotype prediction from casework MPS data.

#### Part C: Forensic DNA phenotyping applications

For the third part of the VISAGE survey on the use of MPS technology for FDP among forensic practitioners in casework, the participating laboratories were asked five questions about their previous experience with MPS applied to different forensic marker sets, their previous or alternative methodology used for SNP genotyping and DNA methylation analysis, and their experience in applying forensic DNA phenotyping to real crime cases.

##### Previous or planned experience of different forensic applications with MPS

The previous MPS experience of all contributing labs was quite diverse for various forensic applications (Table 3; S12). One-third had already gathered knowledge on STR sequencing, identity, and ancestry SNPs, while about one-fifth had previously sequenced mitochondrial DNA or appearance SNPs. The least widespread experience was observed for DNA methylation analysis and mRNA/cDNA sequencing with less than 15%. Nevertheless, between 23 and 40% of participants are planning to explore all of the abovementioned forensic applications by MPS, which shows the overall very high interest in using MPS for the whole toolbox of forensic MPS markers and applications.

**Table 3:** Breakdown of MPS-based forensic genomic applications according to their organizational structure

Type of laboratory	Already done/planned to do	MPS-based forensic application						
(No. of labs)		STR analysis	mtDNA	Identity SNPs	Ancestry SNPs	Appearance SNPs	Methylation analysis	mRNA analysis
Police/governmental	Yes	14	9	12	11	11	4	3
(*n* = 43)	Planned	18	12	20	16	19	21	15
Academic	Yes	13	9	13	14	10	8	7
(*n* = 41)	Planned	16	13	12	11	14	18	10
Private	Yes	5	2	4	2	1	2	1
(*n* = 18)	Planned	5	6	6	6	6	3	2
Other	Yes	2	0	2	1	1	1	1
(*n* = 3)	Planned	0	2	0	1	0	0	0

STR sequencing is the most popular application already carried out at present (32.4%). This is not surprising given that capillary-based STR typing is the standard method of all forensic DNA labs. On the other hand, bisulfite sequencing for DNA methylation analysis, which can be used for age prediction and body fluid identification, is at the top of the list of planned applications with 40%. Apparently it has been realized that this methodology offers very specific data about an unknown stain contributor that can also be searched by the police to reduce a pool of suspects. However, the NGS-based application is hampered by the fact that there are no commercial kits available yet from platform manufacturers for sequencing body fluid-specific methylation markers in a forensic context. Thus, these have to be established and validated based on user applications [6].

When current and planned applications are combined, STR sequencing is the most widely used application (69.5%), followed by identity SNP typing (65.7%), appearance SNP typing (59.1%), and bisulfite sequencing for methylation analysis (54.3%). Notably, interest in ancestry SNP typing was only expressed by 49.5% of the participants. This might be due to the fact that ancestry is more controversial compared to pigmentation traits among the FDP applications, given that there are critical discussions in the public about the risk of discriminating against minorities [4], and thus has not generated as much interest as appearance SNP typing and methylation analysis.

Furthermore, there are no differences regarding previous experience and plans for implementation when the organizational background of the participating laboratories is broken down (Table 3). All MPS-based applications appear to be of similar interest for police/governmental and academic laboratories.

##### Previous experience with SNP genotyping or bisulfite sequencing with conventional methods

In another series of questions, participants were asked for details about the type of SNP markers they had previously investigated and which conventional alternative methods to MPS they had already used for SNP genotyping, or bisulfite sequencing for DNA methylation analysis.

S13 summarizes the percentages of labs using conventional methods based on capillary electrophoresis. Most forensic labs were using a single base primer extension typing which can be done based on a commercial kit termed SNaPshot™ (Thermo Fisher Scientific). Numerous publications are available describing this approach for identity, ancestry, and pigmentation SNP typing [21–24]. Other methods used include Sanger sequencing or restriction fragment length polymorphism (RFLP) analysis, real-time PCR, or SNP genotyping by MALDI-TOF mass spectrometry. S14 provides details about phenotypic markers already explored using conventional methods. Here, the top marker types were appearance, identity, and Y-chromosomal SNPs.

Only 40% of the participants had previous experience with alternative methods in respect to bisulfite sequencing for methylation analysis. This applied mainly to pyrosequencing and single base extension assays analyzed by capillary electrophoresis (S15).

Other methylation sequencing methods applied included the EpiTYPER system (performed by 3 labs), Sanger sequencing, methylation-specific restriction enzyme (MSRE) analysis, real-time PCR, methylation-specific PCR, and multiplex ligation-dependent probe amplification (MLPA) [25, 26].

##### Experience with DNA phenotyping in forensic casework

The final question of this section addressed previous casework experience using forensic DNA phenotyping either regarding criminal investigations or for the identification of unidentified human remains (S16). Surprisingly, 11–20% of participants have already used various types of appearance SNPs, and 23.9% have used ancestry SNPs. In contrast, only 7.6% have used methylation analysis for age prediction. Other predictive markers used included Y-chromosomal and mitochondrial DNA SNPs as well as Y-STR haplotyping.

Between 15 and 21% of the participants reported that predictive markers were not used due to the fact that their application is not legally permitted in their respective country of residence.

#### Part D: Education and training

Part D of the VISAGE survey on the use of MPS technology for forensic DNA phenotyping among forensic practitioners in casework laboratories aimed to identify training needs and priorities for a successful implementation of forensic DNA phenotyping in routine casework.

##### Previous educational workshop and training experiences

First, information on previous training experiences among the participants was collected (S17). Almost 55% of participants have already taken part in various types of training workshops on the general use of MPS technology, most likely in the context of platform-specific training offered by the respective manufacturer. A total of 36% have received no training whatsoever, and 21–30% have already attended more general training on forensic SNP markers, e.g. user workshops in association with scientific meetings such as the biannual congresses of the International Society for Forensic Genetics (ISFG) [27]. Less than 15% have been trained on analysis and interpretation of FDP markers, indicating an urgent need for more educational workshops on the practical application of specific forensic markers and their interpretation in the context of casework.

##### Interest in future educational workshop and training events

As a consequence of the abovementioned lack of training in regard to analysis and interpretation of FDP markers, a high interest for all SNP typing applications was expressed by 43–57% of participants (S18). Furthermore, almost 70% expressed high interest in training on the analysis and interpretation of DNA phenotyping data for predicting appearance, ancestry, and/or age. When high and medium-level interests are combined, then 70–90% of participants have expressed their interest to attend training on one or all of the suggested topics.

#### Part E: Final comments

At the end of the questionnaire, participants had the opportunity to add personal comments or make suggestions relevant for this survey or regarding forensic DNA phenotyping applications in general. A number of participants expressed their strong interest to establish an information exchange regarding casework experiences, validation of MPS-based methods, specific software training, the legal situation in other countries, and the availability of proficiency tests which are essential for obtaining accreditation for these new technologies. Regarding the latter, the German DNA Profiling (GEDNAP) proficiency testing system has recently introduced scientific proficiency test modules for pigmentation markers and age estimation based on blood samples [12].

## Conclusions

This survey has provided an overview on the state-of-the-art of MPS-based DNA analysis and forensic DNA phenotyping among forensic practitioners in Europe. The main outcomes of this survey are summarized below:

1. Availability of MPS platforms
  A total of 73% of the 105 participants from 32 European countries already own an MPS platform or are planning to acquire one within the next 1-2 years. Also, more than 50% of the participants are using more than one software tool to analyze MPS data.
2. Dedicated MPS personnel
  Awareness exists regarding the need for specialized personnel for the operation of MPS technology. A total of 40-50% report on the presence of dedicated technicians and scientists, whereas a bioinformatics expert is only available in 20% of the laboratories.
3. Previous MPS experience
  One-third of the participants have already carried out MPS-based STR sequencing, identity, or ancestry SNP typing. A total of 23-40% of participants are planning to explore all FDP applications showing the overall very high interest in using MPS for the whole range of forensic MPS markers and applications.
4. Previous experience with applying FDP in casework
  Approx. 50% of the participants have previously gathered experience using FDP markers based on conventional (i.e., not MPS-based) DNA typing methods. Already 11-20% of participants have used appearance SNPs and 24% have used ancestry SNPs either in criminal or in identification cases.
5. Training experience
  A total of 55% of the participants have attended training on the general use of MPS technology, but 36% have received no training whatsoever. Less than 15% have been trained on analysis and interpretation for FDP markers, indicating an urgent need for training on the specific forensic markers and their interpretation.
6. Training interest
  Accordingly, 90% have expressed high or medium interest to attend training on the analysis and interpretation of DNA phenotyping data for predicting appearance, ancestry, and age.

Our data confirm findings from a previous survey carried out in 2016 by the DNASeqEx Consortium [9, 28]. The survey received answers from 33 ENFSI DNA Working Group laboratories. A total of 60% of the participants already owned an MPS platform or were planning to purchase one within the next 2 years, i.e. by the end of 2018. The applications of MPS-based DNA sequence analysis included identity SNPs (82%), ancestry SNPs (65%), and autosomal STRs (65%). The participants also expressed concerns about a lack of standardization and missing population genetic data in particular regarding STR sequence analysis, which was not the focus of our survey.

We conclude that our findings will be of great help to develop curricula and contents for new educational workshops addressing the specific needs of forensic geneticists aiming to introduce massively parallel sequencing applications for forensic DNA phenotyping, STR sequencing, age prediction, and body fluid analysis. Such workshops can be organized by both the commercial manufacturers of equipment and biochemical reagent kits, and the scientific community in the context of user-specific training at the national level, at summer schools as well as international congresses [27, 29].

## Supplementary Information

Below is the link to the electronic supplementary material.

Supplementary file1 (PDF 1710 KB)

Supplementary file2 (PDF 274 KB)
