# Supplementary material for: Progress in the implementation of massively parallel sequencing for forensic genetics: results of a European-wide survey among professional users
Source: Int J Legal Med. 2021 Apr 13;135(4):1425–32. doi: 10.1007/s00414-021-02569-0 (PMC8205868; doi:10.1007/s00414-021-02569-0)
Supplement: Supplementary file 1 — Supplementary file1 (PDF 1710 KB) [file 414_2021_2569_MOESM1_ESM.pdf]

VISAGE

VISIBLE ATTRIBUTES THROUGH GENOMICS

Forensic DNA Phenotyping Survey

Inquiry on forensic MPS technology and applications

0 %

General information about this survey

This survey is part of the EU-funded **VISAGE research project** http://www.visage-h2020.eu aiming to develop toolkits suitable for forensic DNA phenotyping using massively parallel sequencing (MPS) platforms, also termed next generation sequencing (NGS).

The purpose of this **survey among forensic practitioners** is to obtain data on the use of MPS platforms in forensic casework laboratories in general, as well as to understand the level of individual knowledge about the genetic markers used for DNA phenotyping, i.e. for the **prediction of appearance (eye, hair and skin color), biogeographic ancestry and age,** as well as their interpretation. Based on the results we will develop training curricula for the technical implementation of DNA phenotyping toolkits. VISAGE will offer **train-the-trainers workshops** to facilitate establishing forensic DNA phenotyping using MPS platforms in forensic genetic laboratories.

You will need approx. 10 minutes for answering the questions of this survey. *You can interrupt the survey at any time. To continue later, please note the individual code which will be given to you!*

Privacy and data protection information: In the context of this survey, we also collect some personal data, such as your educational background and employment status, as well as your email address (optionally; it is required only if you would like to receive a copy of the final report). We will use your data to prepare an anonymized statistical compilation of all results, serving as an overview on the use of MPS in the context of forensic DNA phenotyping applications in forensic genetics. We will never share individual-level data with any third parties outside the VISAGE Consortium. We securely store these data within a protected server environment and will delete the individual datasets one year after the end of the VISAGE project's funding period in April 2021. By participating in this survey and providing your data you consent to our using your data in this manner. In case of questions, please contact the VISAGE Work Package 7 manager Peter Schneider (Institute of Legal Medicine, University Clinic of Cologne) via: visage.wp7@gmail.com .

VISAGE is funded by the EU Horizon 2020 "Secure Societies" Programme (grant agreement no. 740580).

Next

Forensic DNA Phenotyping Survey

Inquiry on forensic MPS technology and applications

7 %

A. Background information

1. Your organisation *

- Police/governmental laboratory
- Academic/university laboratory
- Private laboratory
- Other, please specify

Is your laboratory a member of the ENFSI DNA Working Group? *

- Yes
- No

2. Your country of residence *

Please select

Previous Next

VISAGE
VISIBLE ATTRIBUTES THROUGH GENOMICS

Forensic DNA Phenotyping Survey

Inquiry on forensic MPS technology and applications

14 %

A. Background information

3. Does your laboratory routinely perform forensic DNA typing in the context of criminal investigations?

- ○ Yes
- ○ No

4. Please estimate the percentage of criminal casework in your laboratory

- ○ 100%
- ○ Equal to or more than 50%
- ○ Less than 50%
- ○ No criminal casework performed

Previous Next

Forensic DNA Phenotyping Survey

Inquiry on forensic MPS technology and applications

21 %

A. Background information

5. Your position

- ○ Lab supervisor / director
- ○ Reporting officer / scientist
- ○ Technical assistant
- ○ Other, please specify ______

6. Your education

- ○ BSc
- ○ MSc
- ○ PhD / MD
- ○ Other, please specify ______

Previous | Next

VISAGE
VISIBLE ATTRIBUTES THROUGH GENOMICS

Forensic DNA Phenotyping Survey

Inquiry on forensic MPS technology and applications

29 %

B. Equipment / personnel

1. Does your laboratory own a MPS platform?

- ◯ Yes, for more than 2 years
- ◯ Yes, for less than 2 years
- ◯ No, but a MPS platform will be installed within the next 12 months
- ◯ No, but we are planning to purchase a MPS platform within the next 1-2 years
- ◯ No, and there are no plans for purchasing a MPS platform

Previous | Next

Forensic DNA Phenotyping Survey

VISAGE
VISIBLE ATTRIBUTES THROUGH GENOMICS

Inquiry on forensic MPS technology and applications

36 %

B. Equipment / personnel

2. If you own a MPS platform (or have access), please provide information about the type/model

Please check all that apply

- [ ] No MPS platform available
- [ ] Illumina MiSeq
- [ ] Illumina MiSeq FGx
- [ ] Thermo Fisher Scientific Ion PGM
- [ ] Thermo Fisher Scientific Ion S5 / Gene Studio S5
- [ ] Other MPS platform, please specify ______

3. Please provide information about MPS accessory equipment

Please check all that apply

- [ ] No accessory equipment
- [ ] Robotic platform for library preparation (any brand)
- [ ] Thermo Fisher Scientific Ion Chef
- [ ] Dedicated MPS data / backup network attached storage (NAS) system (any brand)
- [ ] Other, please specify ______

VISAGE
VISIBLE ATTRIBUTES THROUGH GENOMICS

Forensic DNA Phenotyping Survey

Inquiry on forensic MPS technology and applications

43 %

B. Equipment / personnel

4. Which type of software, data analysis or interpretation tools are you using for forensic MPS data?

Please check all that apply

- ☐ Not applicable
- ☐ Software provided by platform manufacturer
- ☐ In-house bioinformatic tools
- ☐ Online interpretation resources, e.g. SNIPPER, HIrisplex-S
- ☐ Open source or other software, please specify ____________

Previous | Next

VISAGE
VISIBLE ATTRIBUTES THROUGH GENOMICS

Forensic DNA Phenotyping Survey

Inquiry on forensic MPS technology and applications

50 %

B. Equipment / personnel

5. Do you already have (or plan to have) dedicated personnel for operating the MPS platform?

Please check all that apply

	Library preparation	SNP data analysis	Phenotype prediction	No / not planned
Technical assistant	☐	☐	☐	☐
Bioinformatics expert	☐	☐	☐	☐
Scientist (molecular genetics)	☐	☐	☐	☐

Previous | Next

Forensic DNA Phenotyping Survey

VISAGE
VISIBLE ATTRIBUTES THROUGH GENOMICS

Inquiry on forensic MPS technology and applications

57 %

C. Forensic DNA phenotyping applications

1. Which forensic applications has your laboratory already explored by MPS, or plans to explore?

	Already done	Planning to do	Not interested
STR sequencing	○	○	○
mtDNA sequencing	○	○	○
Identity SNP typing	○	○	○
Ancestry SNP typing	○	○	○
Appearance SNP typing	○	○	○
Bisulfite sequencing for methylation analysis	○	○	○
mRNA/cDNA sequencing	○	○	○

Previous   Next

VISAGE
VISIBLE ATTRIBUTES THROUGH GENOMICS

Forensic DNA Phenotyping Survey

Inquiry on forensic MPS technology and applications

64 %

C. Forensic DNA phenotyping applications

2. Do you have previous practical experience with SNP genotyping (not using MPS)?

- ◯ No
- ◯ Yes, single base extension sequencing using capillary electrophoresis (SNaPshot, SNuPE)
- ◯ Yes, other (please specify) ______

3. Which types of SNP markers have you already investigated using conventional methods?

Please check all that apply!

- ☐ Not applicable
- ☐ Identity SNPs
- ☐ Y-chromosomal SNPs
- ☐ Appearance SNPs (e.g. Irisplex)
- ☐ Ancestry SNPs
- ☐ Other, please specify ______

Previous | Next

VISAGE
VISIBLE ATTRIBUTES THROUGH GENOMICS

Forensic DNA Phenotyping Survey

Inquiry on forensic MPS technology and applications

71 %

C. Forensic DNA phenotyping applications

4. Regarding DNA methylation analysis, have you already used other methods for bisulfite sequencing?

Please check all that apply

- [ ] No
- [ ] Yes, pyrosequencing
- [ ] Yes, single base extension sequencing using capillary electrophoresis (SNaPshot, SNuPE)
- [ ] Yes, other (please specify) ____________

5. Do you already have forensic casework experience with DNA phenotyping (either for criminal cases or for unidentified human remains)?

(You can add more items/lines, if needed)

	Already done	Not done	Not permitted	Not applicable
Appearance SNPs: eye color	○	○	○	○
Appearance SNPs: hair color	○	○	○	○
Appearance SNPs: skin color	○	○	○	○
Ancestry-informative SNPs	○	○	○	○
DNA methylation analysis for age prediction	○	○	○	○
Other, please specify ____________	○	○	○	○

+1

VISAGE
VISIBLE ATTRIBUTES THROUGH GENOMICS

Forensic DNA Phenotyping Survey

Inquiry on forensic MPS technology and applications

79 %

D. Education & training

1. Have you already attended a training or educational workshop on the following topics?

Please check all that apply

- [ ] General MPS data analysis
- [ ] Introduction to Identity SNP typing
- [ ] Introduction to Ancestry SNP typing
- [ ] Introduction to Appearance SNP typing
- [ ] Bisulfite sequencing for methylation analysis
- [ ] Analysis & interpretation of DNA phenotyping data for predicting appearance, ancestry and/or age
- [ ] None

Previous Next

VISAGE
VISIBLE ATTRIBUTES THROUGH GENOMICS

Forensic DNA Phenotyping Survey

Inquiry on forensic MPS technology and applications

88 %

D. Education & training

2. Please indicate your interest to attend a workshop on the following topics:

	High	Medium	Low	No interest
General MPS data analysis	○	○	○	○
Introduction to Identity SNP typing	○	○	○	○
Introduction to Ancestry SNP typing	○	○	○	○
Introduction to Appearance SNP typing	○	○	○	○
Bisulfite sequencing for methylation analysis	○	○	○	○
Analysis & interpretation of DNA phenotyping data for predicting appearance, ancestry and/or age	○	○	○	○

Previous Next

VISAGE
VISIBLE ATTRIBUTES THROUGH GENOMICS

Forensic DNA Phenotyping Survey

Inquiry on forensic MPS technology and applications

93 %

E. Final comments

1. Please add your comments and suggestions, if you feel something is missing in this survey, and any other relevant information:

2. Please add your email address, if you want to receive a copy of the report summarising the results of this survey

Thank you very much for participating in this survey. If you have provided your email address, we will send you the report. To obtain more information abouth the VISAGE project, please visit http://www.visage-h2020.eu!

Previous Done
