# Supplementary material for: Progress in the implementation of massively parallel sequencing for forensic genetics: results of a European-wide survey among professional users
Source: Int J Legal Med. 2021 Apr 13;135(4):1425–32. doi: 10.1007/s00414-021-02569-0 (PMC8205868; doi:10.1007/s00414-021-02569-0)
Supplement: Supplementary file 2 — Supplementary file2 (PDF 274 KB) [file 414_2021_2569_MOESM2_ESM.pdf]

Supplementary Figures

Progress in the implementation of massively parallel sequencing for forensic genetics: results of a European-wide survey among professional users

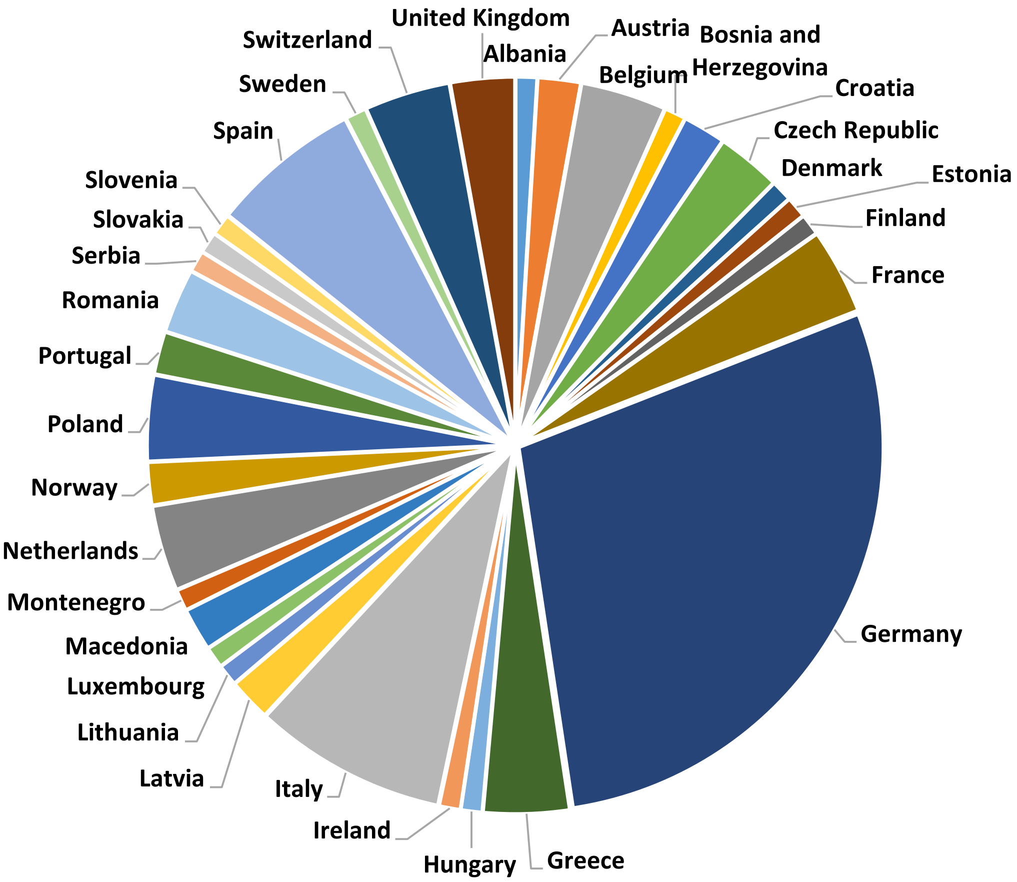

Figure S1: Country of residence of participating laboratories from Europe

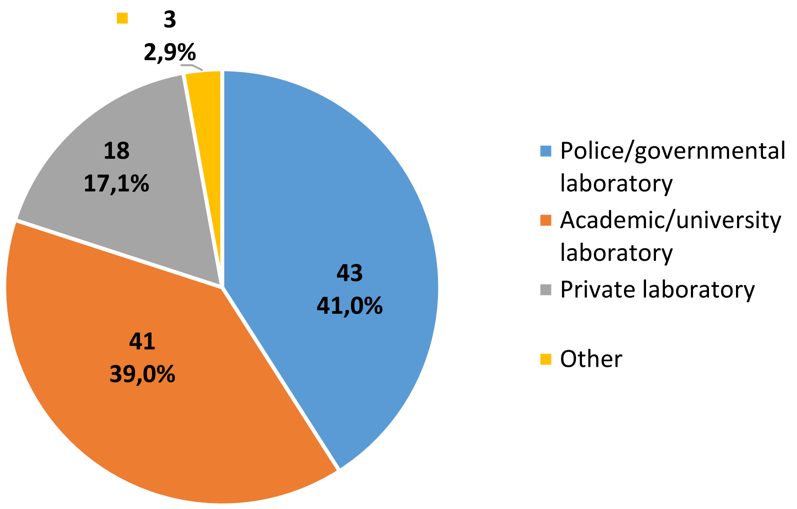

Figure S2: Percentages of European survey participants regarding their organizations and affiliations. The category 'Other' (yellow) comprises international organizations or public or governmental laboratories associated to a university.

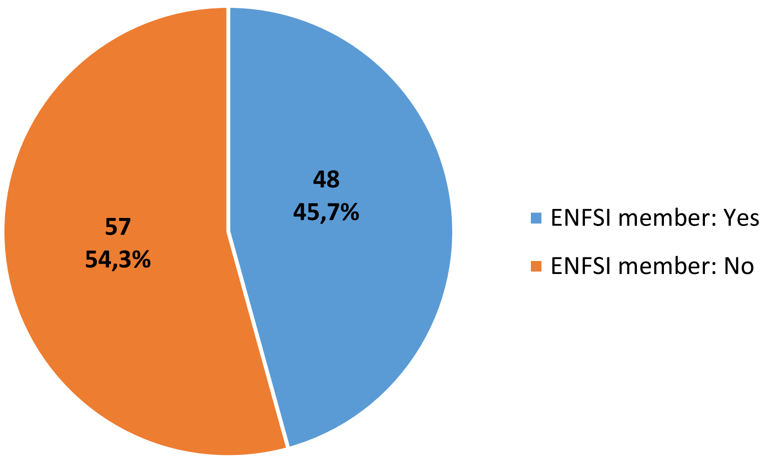

Figure S3: Proportion of European survey participants with a full or associated membership in the ENFSI DNA Working Group

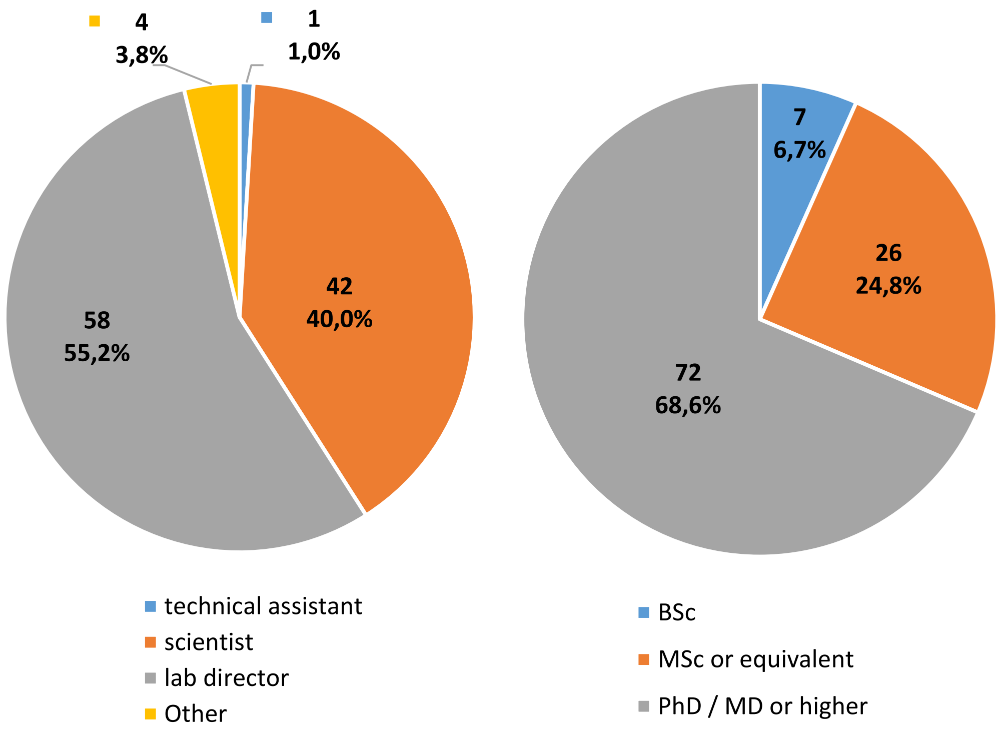

Figure S4: Position (left chart) and education (right chart) of participating employee of each respective lab. The category 'Other' (yellow, left chart) comprises roles such as research and development or quality manager or postdoctoral researcher.

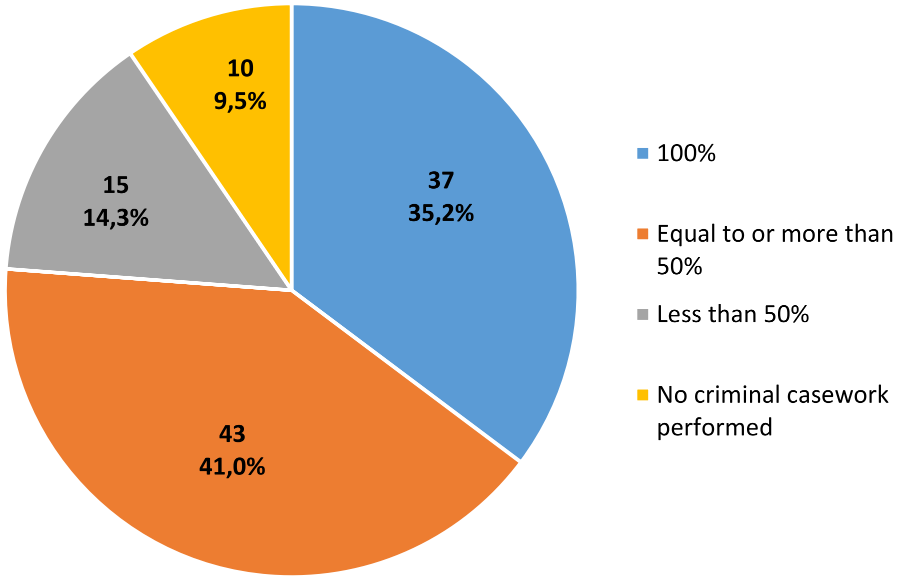

Figure S5: Proportion of criminal casework performed in participating laboratories.

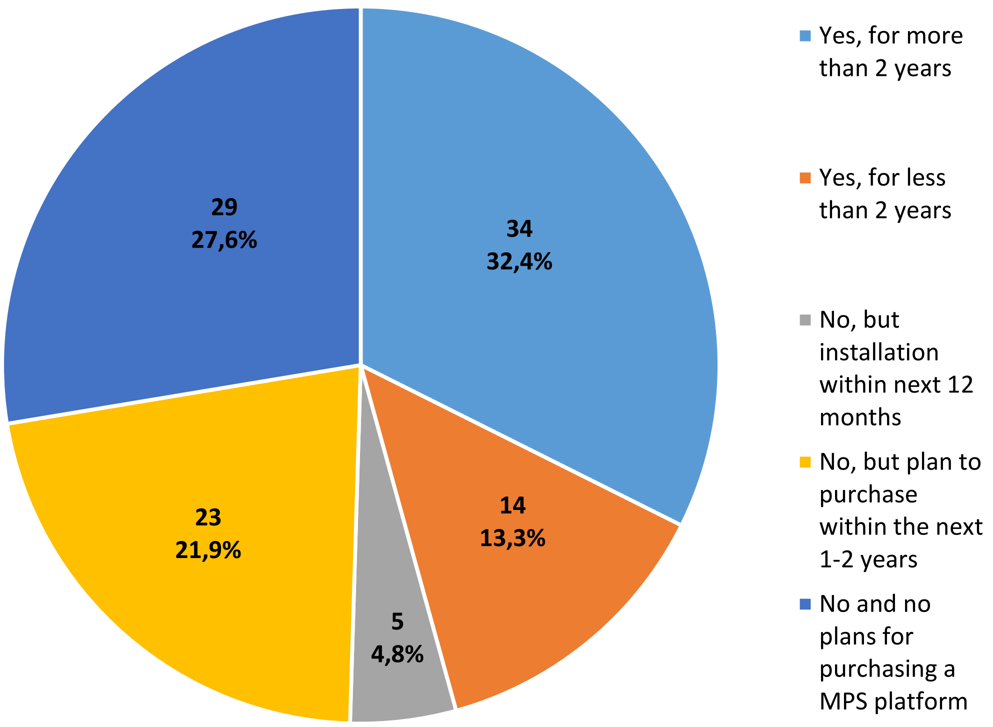

Figure S6: Ownership of MPS platform. The question asked was 'Does your laboratory own a MPS platform?'

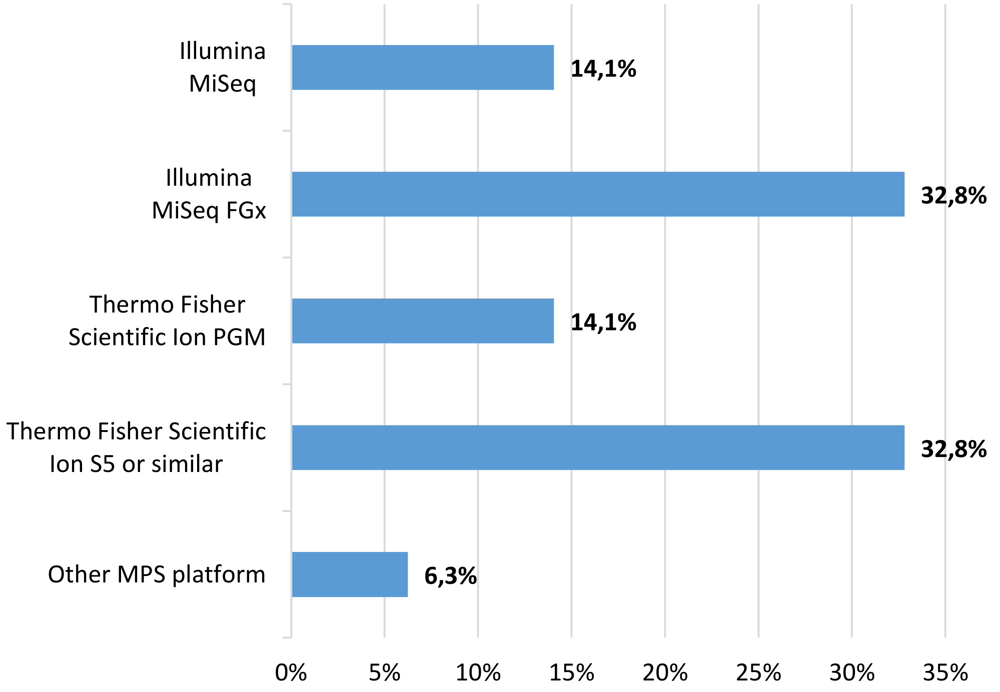

Figure S7: Types and distribution of MPS platforms available in European laboratories (n = 64 instruments = 100%).

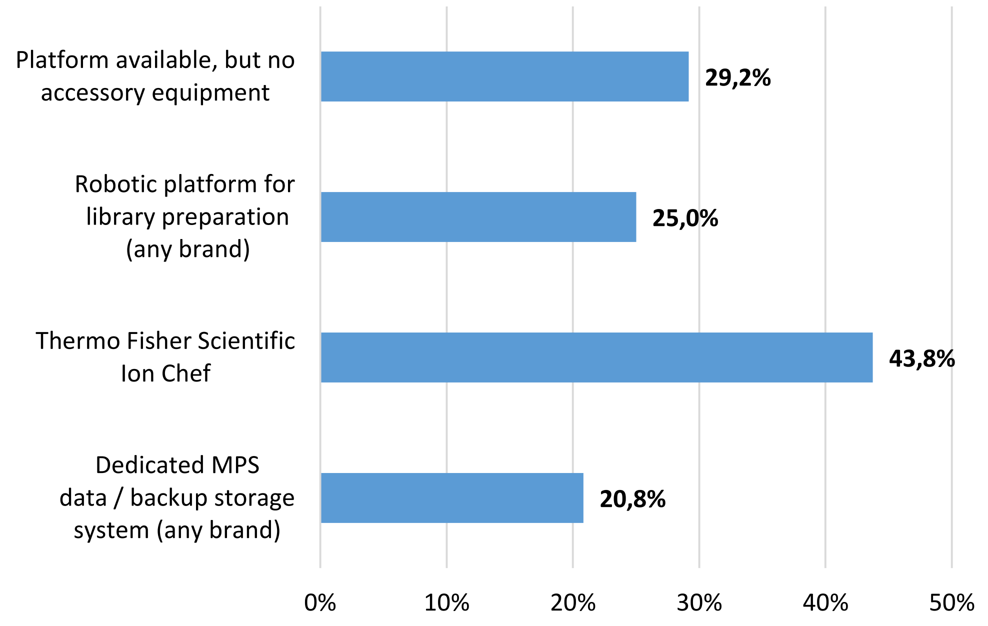

Figure S8: Accessory MPS equipment (n = 48, participants with MPS platform)

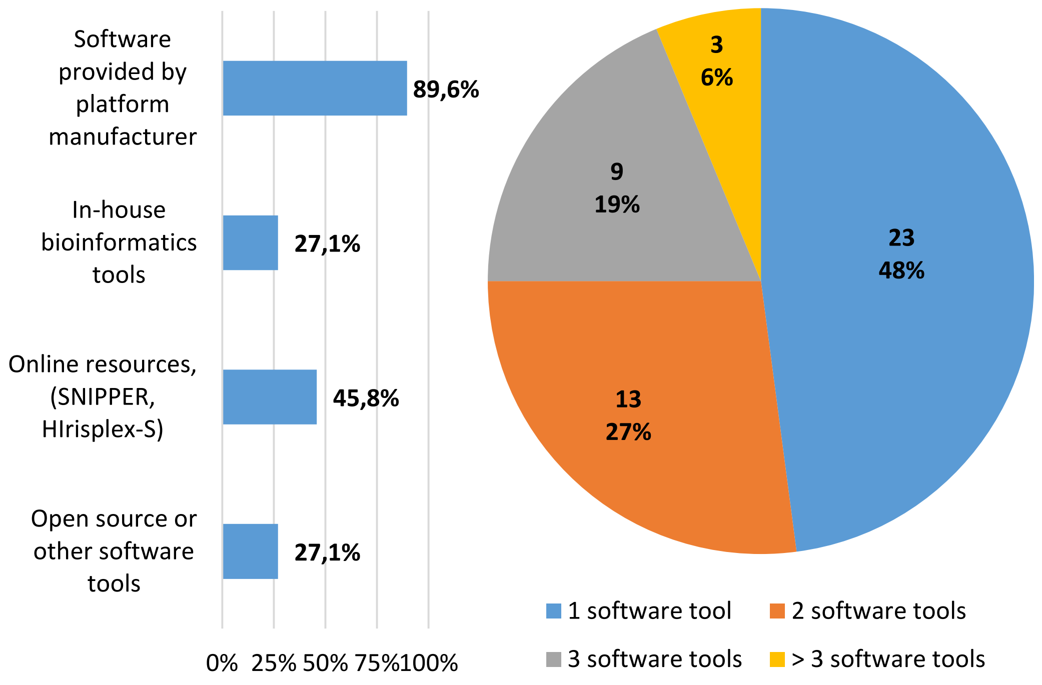

Figure S9: Analysis software used by survey participants

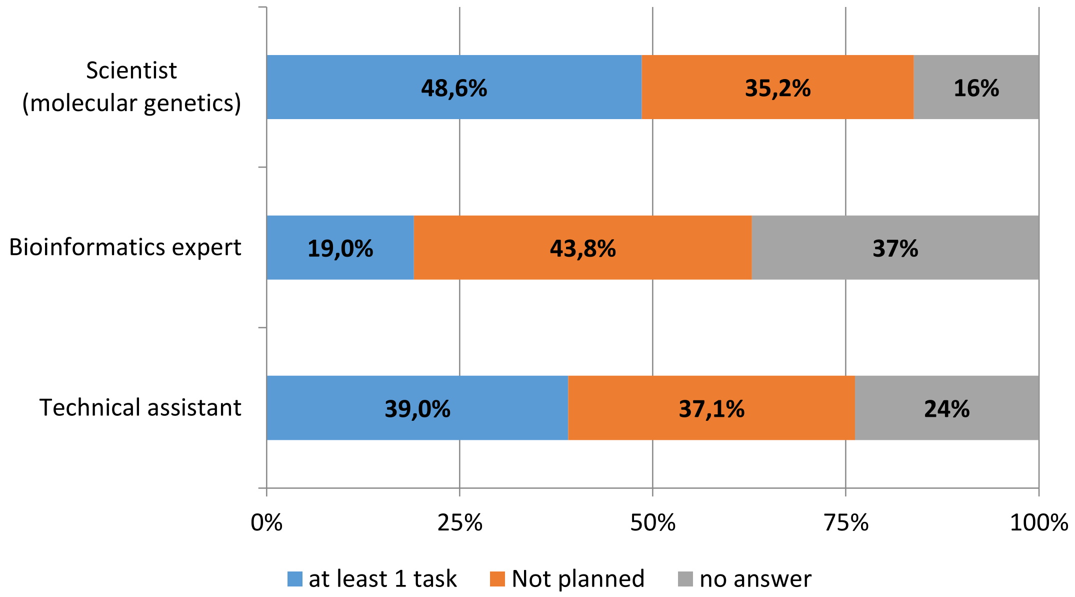

Figure S10: Percentages of different personnel dedicated to operation of MPS library preparations, platforms and data analysis (n=93)

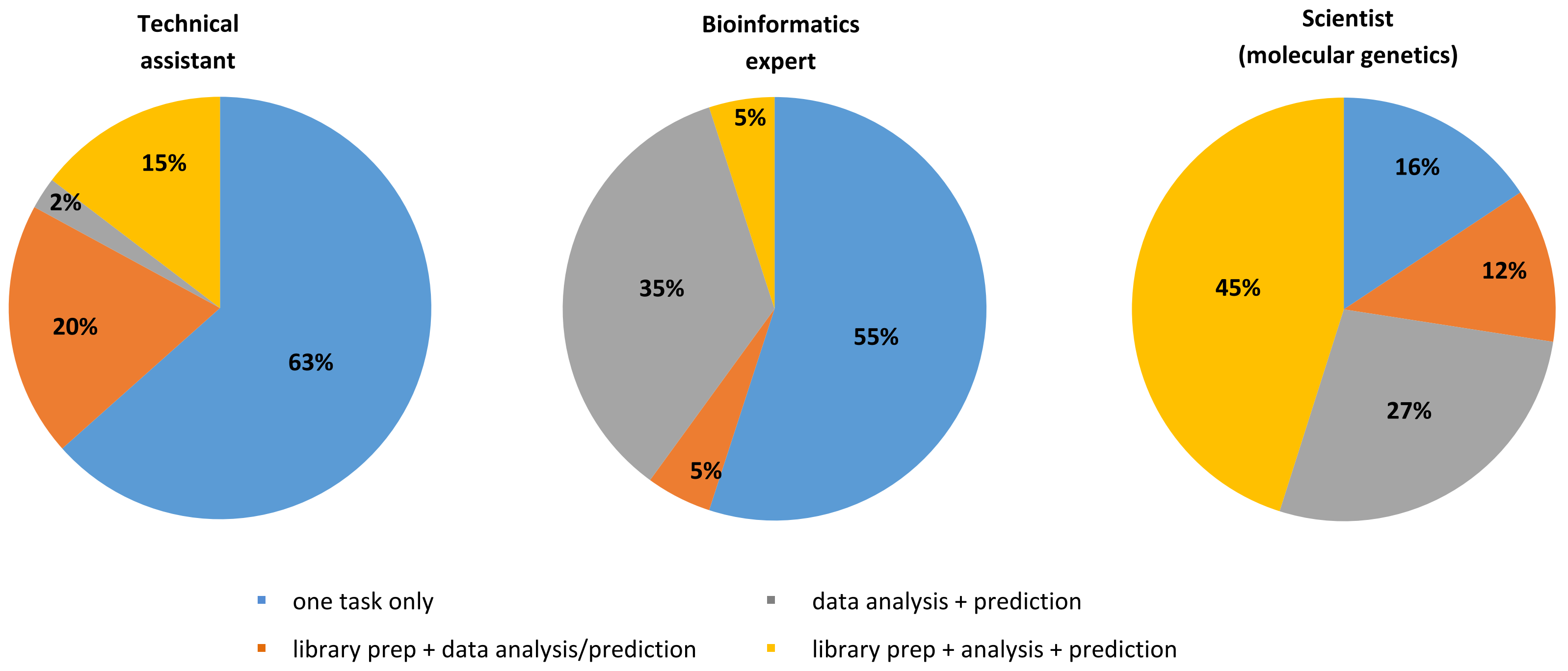

Figure S11: Dedicated personnel for MPS library preparation, data analysis and phenotype prediction.

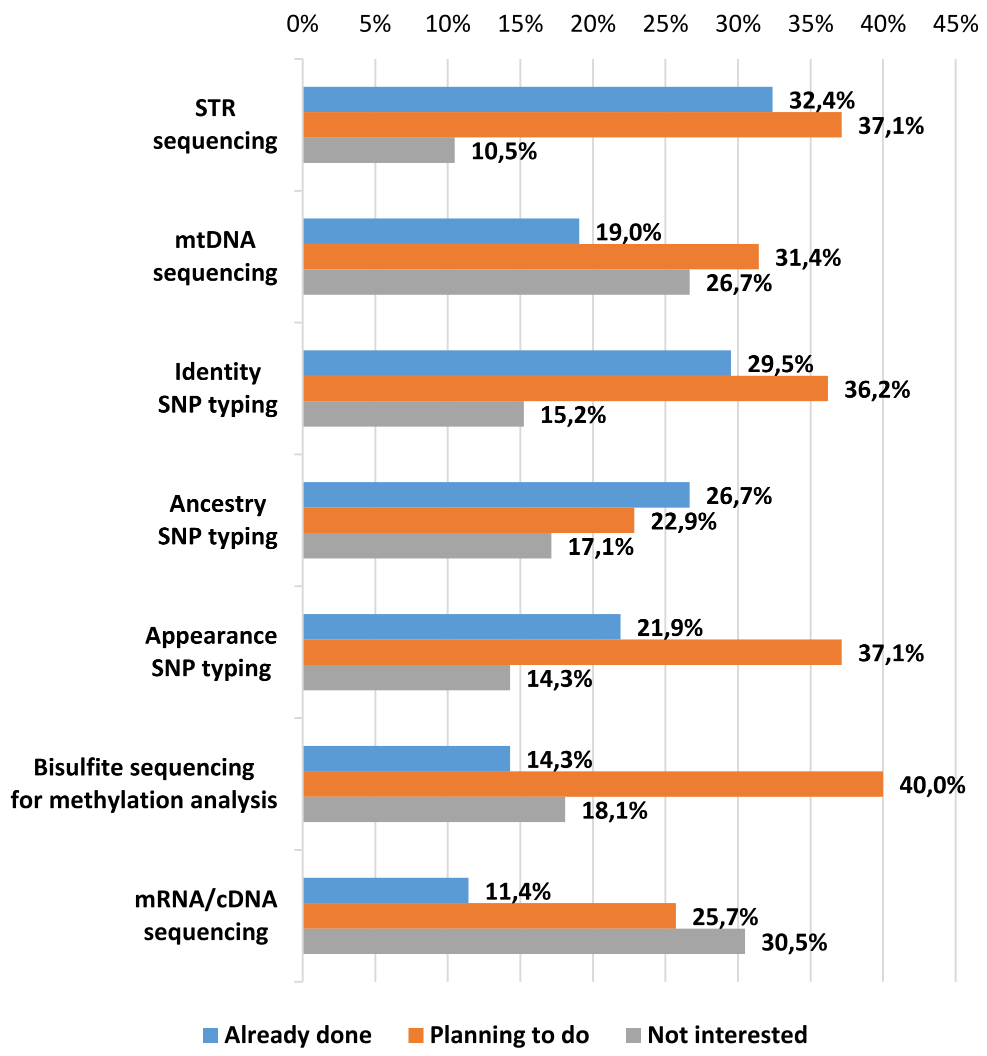

Figure S12: Forensic applications already performed or planned to be explored by MPS in participating laboratories. Missing percentage adding up to 100% per category were 'no replies', e.g. for the first category 'STR sequencing' 20% of labs chose not to tick any answer etc.

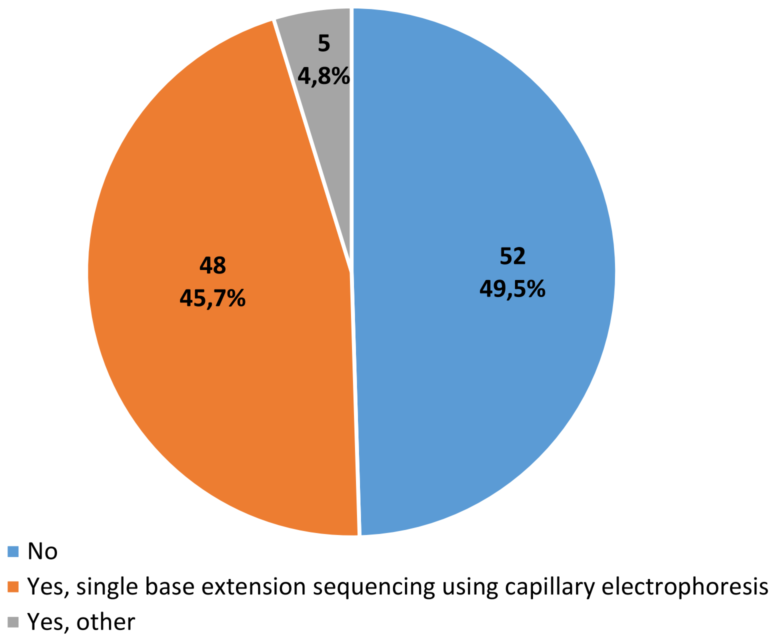

Figure S13: Previous experience with SNP genotyping by conventional methods in all European labs (N =105).

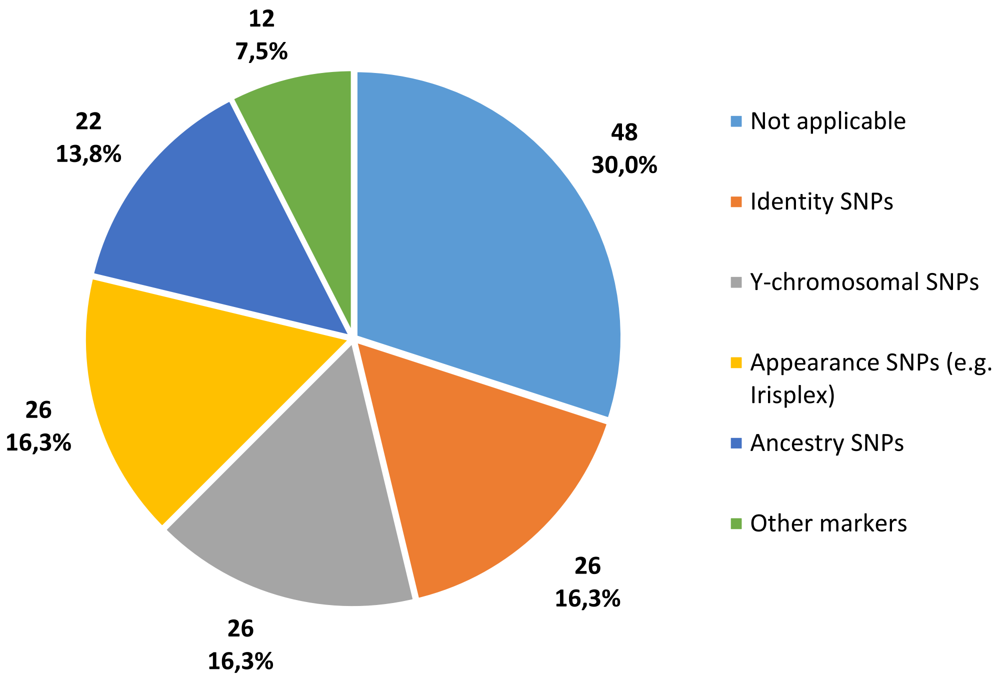

Figure S14: Forensic SNP genotyping applications previously explored by participating laboratories (N = 96, multiple answers possible). The category 'Other markers' comprises mitochondrial DNA SNPs as well as SNPs with relevance for clinical diagnostics.

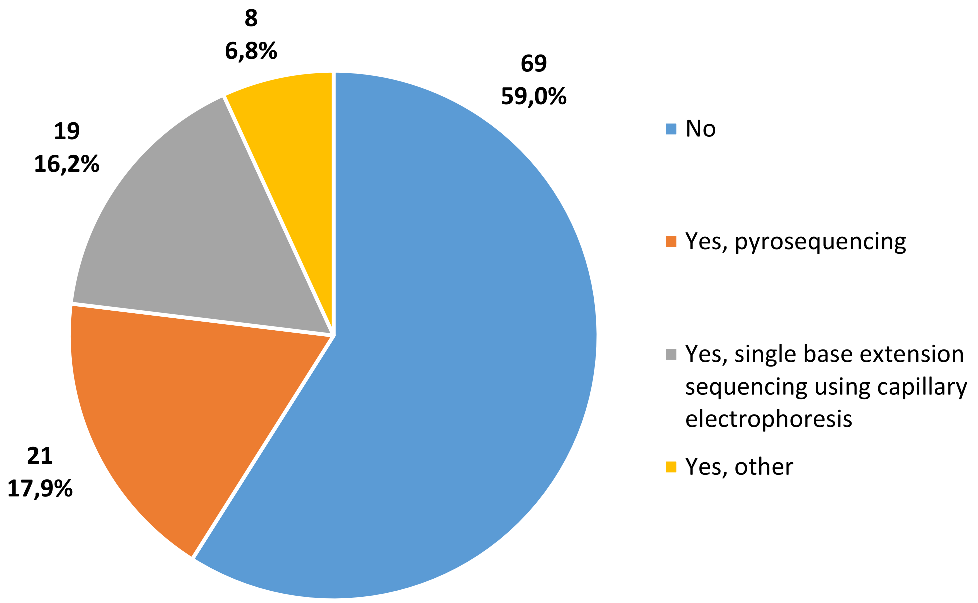

Figure S15: Experience with alternative methods for DNA methylation analysis apart from MPS in participating laboratories (N = 102, multiple answers possible).

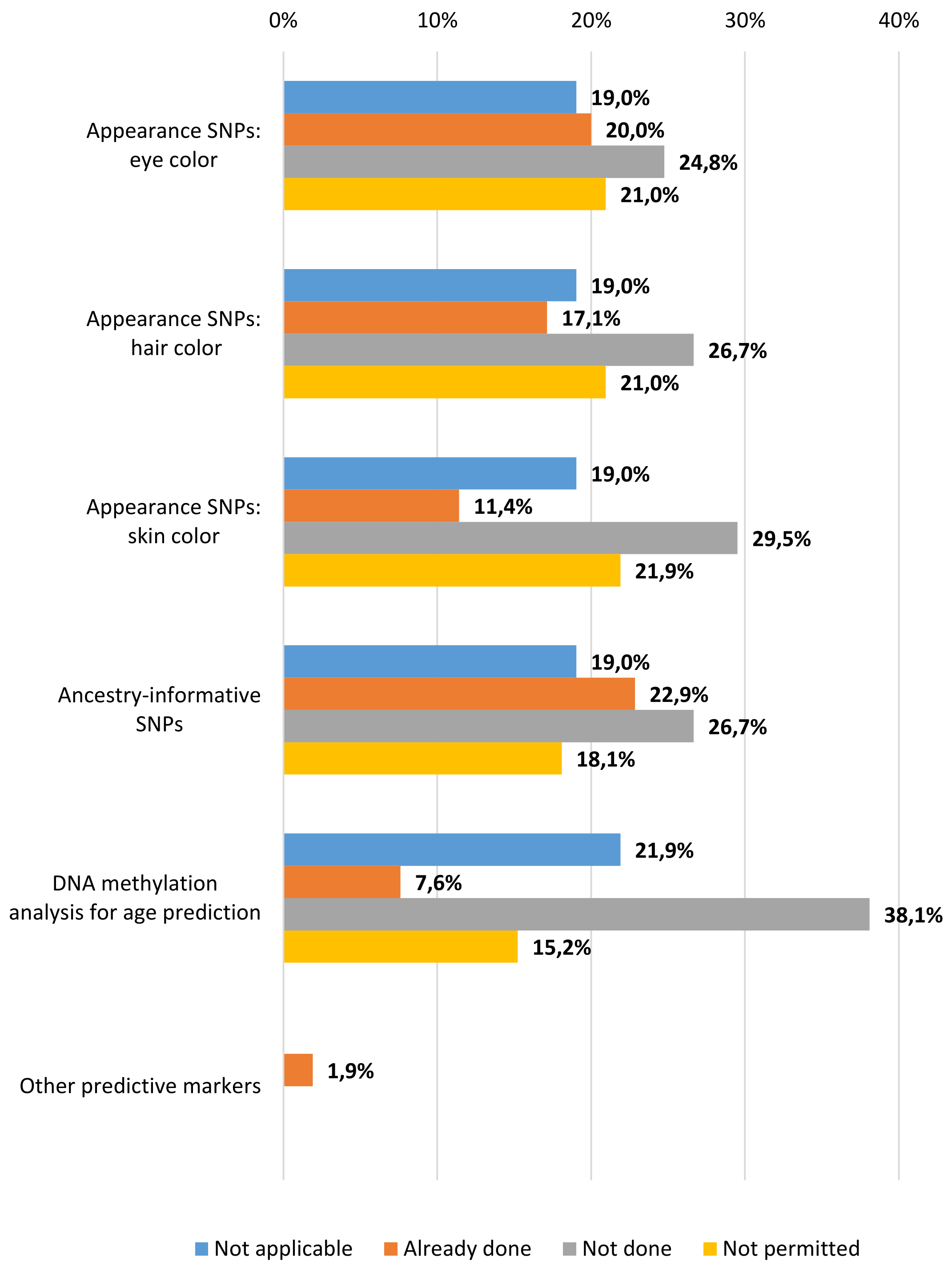

Figure S16: Previous experience with the application of forensic DNA phenotyping in forensic casework (please note that percentages do not add up to 100% as not all participants answered all questions)

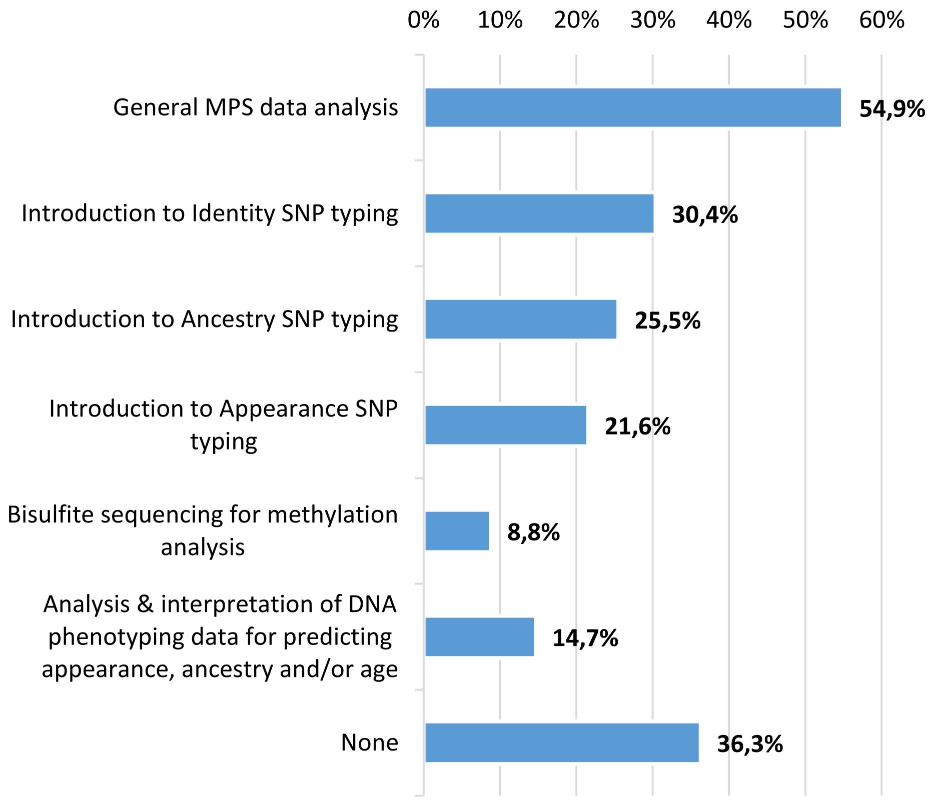

Figure S17: Have you already attended a training or educational workshop on the following topics? (n=102, multiple answers possible)

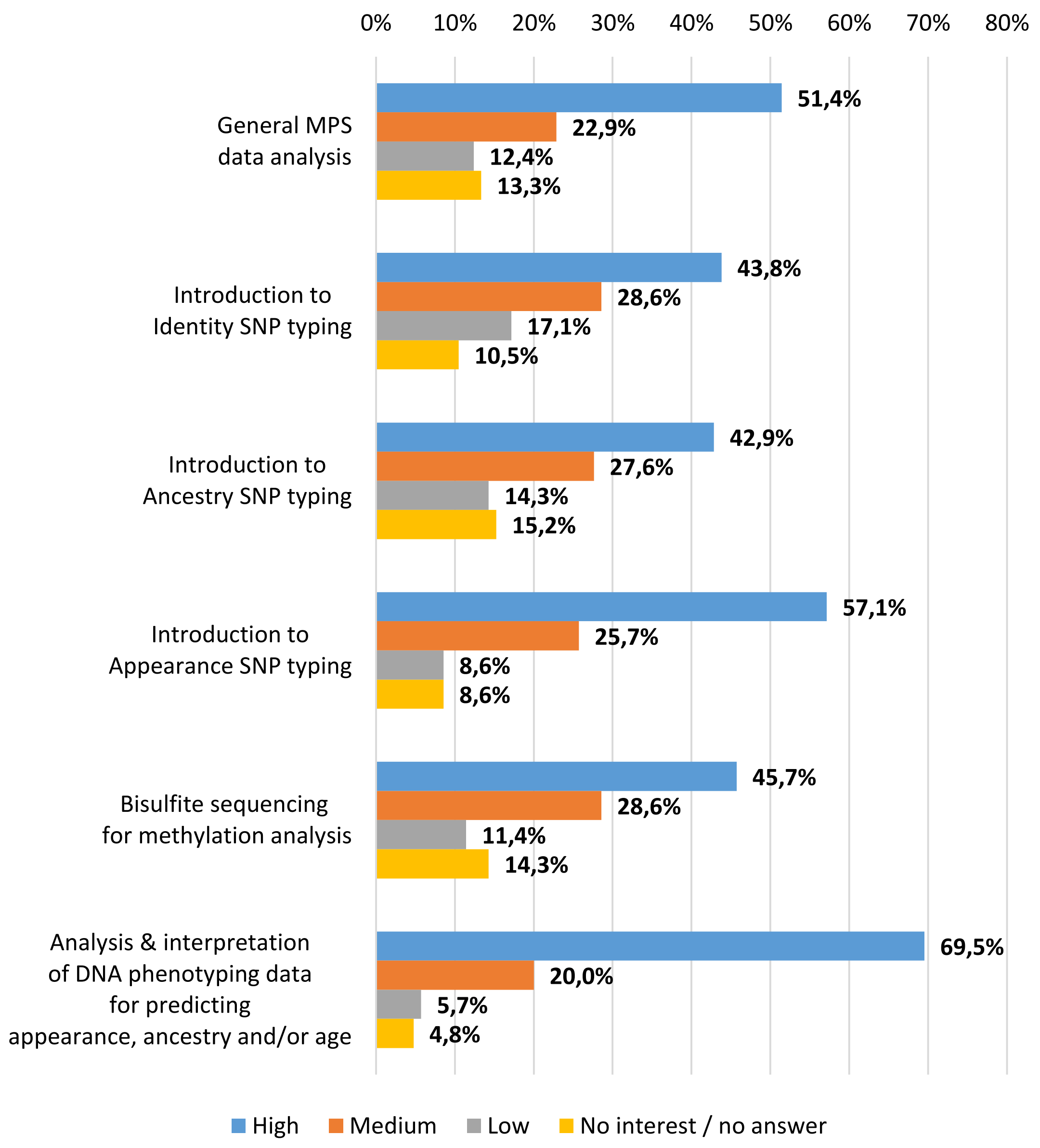

Figure S18: Participating laboratories' interest in training events by topic (N = 105)
